# Testing consumer theory: evidence from a natural field experiment

**DOI:** 10.1007/s40881-017-0040-3

**Published:** 2017-11-28

**Authors:** Maja Adena, Steffen Huck, Imran Rasul

**Affiliations:** 1 WZB, Berlin, Germany; 2 UCL, London, United Kingdom

**Keywords:** Natural field experiment, Revealed preference, C93, D01, D12, D64

## Abstract

We present evidence from a natural field experiment designed to shed light on whether individual behavior is consistent with a neoclassical model of utility maximization subject to budget constraints. We do this through the lens of a field experiment on charitable giving. We find that the behavior of at least 80% of individuals, on both the extensive and intensive margins, can be rationalized within a standard neoclassical choice model in which individuals have preferences, defined over own consumption and their contribution towards the charitable good, satisfying the axioms of revealed preference.

**Electronic supplementary material:**

The online version of this article (10.1007/s40881-017-0040-3) contains supplementary material, which is available to authorized users.

## Introduction

Neoclassical theory provides a rich set of testable implications for how consumer demand responds to changes in relative prices and income. This paper presents evidence from the first large-scale natural field experiment shedding light on whether individual behavior is consistent with the predictions of revealed preference theory within a standard model of utility maximization subject to budget constraints (e.g., Afriat 1967). We do this through the lens of a natural field experiment on charitable giving.

By focusing our analysis on the choice between a charitable good and private consumption, we vary the budget set individuals face in a straightforward and natural way, holding all other prices constant. We do so by offering various *matching schemes *that affect how donations given for the charitable good translate into donations received by the project. Specifically, we induce—(i) large changes in the relative price of the charitable good through rates at which donations are matched; (ii) pure income transfers to individuals through a matching scheme that guarantees *any* positive donation is matched by some fixed amount; (iii) a non-convex budget set in which only donations above some threshold are matched.

In our design, the induced budget sets intersect each other, opening up the possibility to directly test the predictions of revealed preference theory. For such research questions, a between-subject research design is strictly preferred to a within-subject design. This is because within-subject designs inevitably require the same individual to be presented with different budget sets at *different* moments in time. This raises the concern that there are natural changes over time in incomes, relative prices, asset holdings, or labor supplies that confound any inference that can be made on whether individual preferences satisfy the axioms of revealed preference.

Our main result is that on both the extensive and intensive margins of charitable giving, individual choices can be rationalized within a standard model of consumers maximizing utility subject to budget constraints, where individual preferences are defined over own consumption and charitable donations received by the project. The behavior of at least 80% of recipients who make some positive contribution is in line with their preferences satisfying GARP. In short, in a real-world environment where participants make simple decisions they are familiar with, the predictions of microeconomic theory work well in explaining individual behavior.

We highlight that field experiments can be used to test revealed preference theory and such approaches are complementary to non-experimental tests of consumer theory which typically exploit panel data on consumer purchases. However, as in within-subject experimental designs, in non-experimental data apparent violations of revealed preference might instead be due to changes in tastes, changes in the holding of durables, or the storage of consumables and consumption expenditures are typically measured with error. Consumer panels also typically suffer from observed price changes being both relatively small, and not necessarily implying an intersection of budget sets. Hence, in contrast to our research design, tests of revealed preference based on non-experimental data are likely to have low power (Varian 1982; Bronars 1995). Such approaches have provided mixed results with some studies rejecting behavior consistent with GARP (Mossin 1972; Hardle et al. 1991) and others finding more rationalizable patterns of consumption (Manser and Mcdonald 1988, Famulari 1995). Methodological advances using non-parametric techniques suggest that consumer behavior does not reject GARP in the long run for most income groups (Blundell et al. 2003).

Our analysis also builds on laboratory evidence on consumer choice, which has provided mixed evidence on whether individual behavior is consistent with GARP (Battalio et al. 1973; Cox 1997; Sippel 1997; Andreoni and Miller 2002; Choi et al. 2007; List and Lucking-Reiley 2002). Our research design combines the key advantages of laboratory experiments in being able to experimentally manipulate the economic environment faced by agents with the advantages of a field study using real-world data on a large population. As suggested by Varian (2006), this research design is, perhaps, the best possible that could be used to test whether individual behavior is consistent with revealed preference theory.^1^ $$^{,}$$ 2

## The natural field experiment

### Design

In June 2006, the Bavarian State Opera organized a mail out of letters to over 25,000 individuals designed to elicit donations for a social youth project which the opera was engaged in. The project’s beneficiaries are children from disadvantaged families whose parents are almost surely not among the recipients of the mail out. As it is not one large event that donations are sought for, but rather a series of several smaller events, it is clear to potential donors that additional money raised can fund additional activity. In other words, the marginal contribution will *always* make a difference to the project.

Individuals were randomly assigned to one of five treatments that varied in how individual donations would be matched by an anonymous lead donor. The format and wording of the mail out is provided in the Appendix. The mail out letters were identical in all treatments with the exception of one paragraph. Since the presence of a lead donor may serve as a signal of project quality (Vesterlund 2003; Andreoni 2006), it is essential that the lead donor is also mentioned in a baseline treatment. Hence in the control treatment T1, recipients were informed that the project had already garnered a lead gift of €60,000, but there was no offer to match donations. The wording of the key paragraph read as follows:

- T1 (control): a generous donor who prefers not to be named has already been enlisted. He will support “Stück für Stück” with €60,000. Unfortunately, this is not enough to fund the project completely which is why I would be glad if you were to support the project with your donation.
- T2 (50% matching): a generous donor who prefers not to be named has already been enlisted. He will support “Stück für Stück” with up to €60,000 by donating, for each Euro that we receive within the next 4 weeks, another 50 Euro cent. In light of this unique opportunity, I would be glad if you were to support the project with your donation.
- T3 (100% matching): a generous donor who prefers not to be named has already been enlisted. He will support “Stück für Stück” with up to €60,000 by donating, for each donation that we receive within the next 4 weeks, the same amount himself. In light of this unique opportunity, I would be glad if you were to support the project with your donation.
- T4 (non-convex): a generous donor who prefers not to be named has already been enlisted. He will support “Stück für Stück” with up to €60,000 by donating, for each donation above €50 that we receive within the next four weeks, the same amount himself. In light of this unique opportunity, I would be glad if you were to support the project with your donation.
- T5 (income): a generous donor who prefers not to be named has already been enlisted. He will support “Stück fü r Stück” with up to €60,000 by donating, for each donation that we receive within the next 4 weeks regardless of the donation amount, another €20. In light of this unique opportunity, I would be glad if you were to support the project with your donation.

Notice how T4 and T5 generate budget constraints that overlap and cross with others thus generating revealed preference predictions.

### Conceptual framework

We assume that potential donors have preferences defined over two dimensions—their own consumption, *c*, and the marginal benefit their donation provide, $d_{r}$. In our setting, we then have two goods—donations received by the project, and a composite good representing all other consumption. We denote the price and goods vectors as $\mathbf {p}$ and $\mathbf {x}$, respectively. As in the exposition of Varian (2006), we then have the following definitions.

- **Definition (revealed preference)** Given some vector of prices and chosen bundles ($\mathbf {p}^{t},\mathbf {x}^{t}$) for $t=1,\ldots ,T$, $\mathbf {x} ^{t}$ is directly revealed preferred to $\mathbf {x}$ if $\mathbf {p}^{t} \mathbf {x}^{t}\ge \mathbf {p}^{t}\mathbf {x}$. $\mathbf {x}^{t}$ is indirectly revealed preferred to $\mathbf {x}$ if there is some sequence $r,s,t,\ldots ,u,v$, such that $\mathbf {p}^{r}\mathbf {x}^{r}\ge \mathbf {p}^{r}\mathbf {x}^{s},$ $\mathbf {p}^{s}\mathbf {x}^{s}\ge \mathbf {p}^{s}\mathbf {x}^{t},\ldots ,\mathbf {p}^{u}\mathbf {x}^{u}\ge \mathbf {p}^{u}\mathbf {x}$.
- **Definition (weak axiom of revealed preference)** If $\mathbf {x}^{t}$ is directly revealed preferred to $\mathbf {x}^{s}$, then it is not the case that $\mathbf {x}^{s}$ is directly revealed preferred to $\mathbf {x}^{t}$, so that $\mathbf {p}^{t}\mathbf {x}^{t}\ge \mathbf {p}^{t}\mathbf {x}^{s}$ implies that $\mathbf {p}^{s}\mathbf {x}^{s}<\mathbf {p}^{s}\mathbf {x}^{t}$.
- **Definition (generalized axiom of revealed preference)** The data ($\mathbf {p}^{t},\mathbf {x}^{t}$) satisfy the generalized axiom of revealed preference (GARP) if $\mathbf {x}^{t}$ is (directly or indirectly) revealed preferred to $\mathbf {x} ^{s}$ implies that $\mathbf {p}^{s}\mathbf {x}^{s}\le \mathbf {p}^{s}\mathbf {x}^{t}$ .

In two dimensions as in our setting, the Weak and Generalized Axioms of Revealed Preference are equivalent. The main result in the revealed preference literature is from Afriat (1967) which states that given some choice data ($\mathbf {p}^{t},\mathbf {x}^{t}$) for $t=1,\ldots ,T,$ the following conditions are equivalent: (i) the data satisfy GARP; (ii) there exists a non-satiated, continuous, monotone, and concave utility function, $u(\mathbf { x})$ that rationalizes the data. In our setting, this corresponds to individual behavior being rationalized by the following utility maximization problem:

1 $$\begin{aligned} \underset{d_{r}}{\max }\;u(c,d_{r})\text { subject to }\; c+d_{g}\le y,\text { } c,d_{g}\ge 0, \quad \text { and } \quad d_{r}=f(d_{g}), \end{aligned}$$

where $u(c,d_{r})$ has the properties listed above, the first constraint ensures consumption can be no greater than income net of any donation given, $y-d_{g}$, the second constraint requires consumption and donations given to be non-negative, and the third constraint denotes the matching scheme that translates donations given into those received by the opera house.

**Fig. 1:**
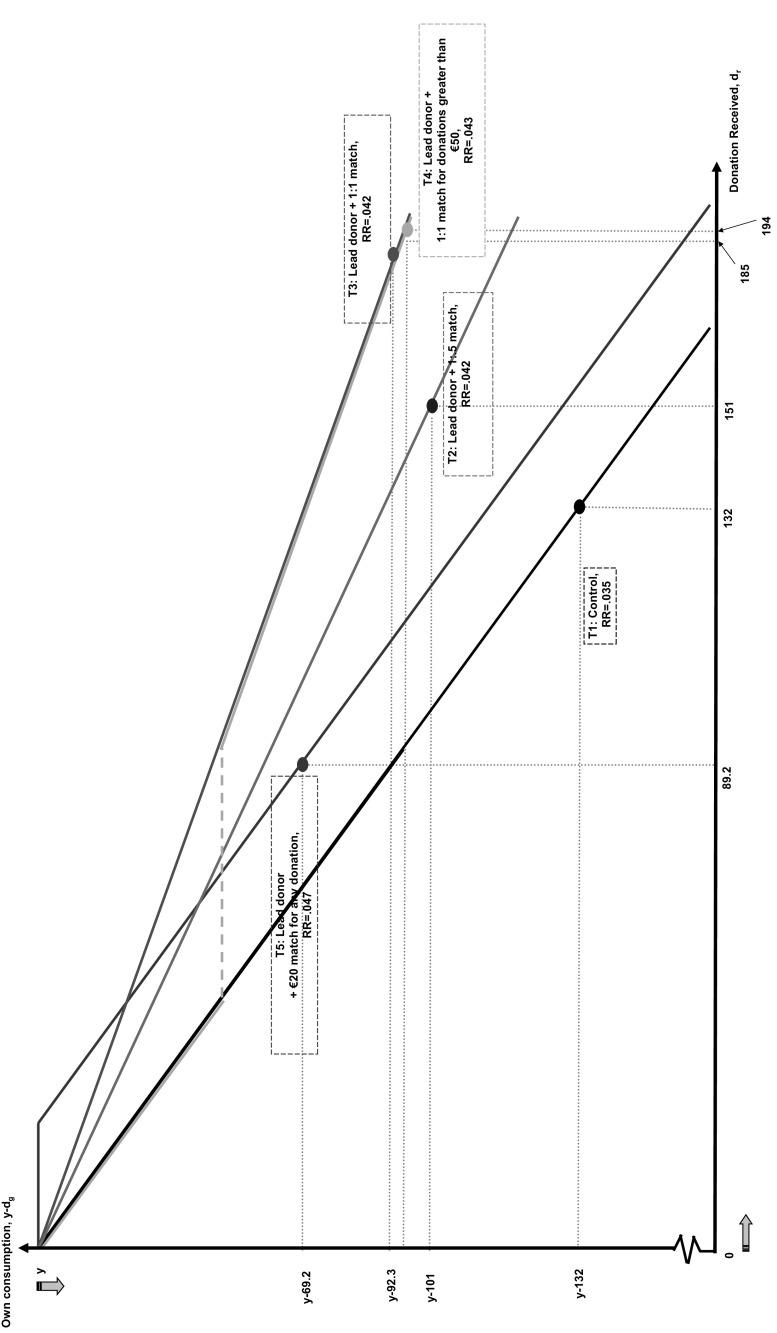
The Design of the Field Experiment and Outcomes by Treatment. **Notes:** This figure graphs the budget sets induced by the five treatments in ($y-d_{g}$, $d_{r}$)-space. The average in each treatment is marked by a dot on a budget line, and the donation received is marked at the horizontal axis, while the donation given is marked at the vertical axis. RR is the response rate in each treatment

Figure 1 graphs the budget sets induced by the five treatments in $(y-d_{g},d_{r})$-space. As the budget sets across treatments intersect, pairwise comparisons of the behavior of individuals in any two treatments allow us to test whether consumer behavior is, on average, consistent with GARP. However, although behavior, on average, might be consistent, each individual’s preferences may violate GARP. We, therefore, exploit the random assignment of recipients to treatments to test for *individual* violations of GARP.

## Descriptives

### Treatment assignment, and extensive and intensive margin outcomes

Table 1 summarizes information on individuals in each treatment and reports the *p* values on the null hypothesis that the mean characteristic of individuals in the treatment group is the same as in the control group T1. There are no significant differences along any dimension between recipients in each treatments.

**Table 1:** Characteristics of recipients by matching treatment

Treatment number	Treatment description	Number of individuals	Female [yes$\,=\,$1]	Number of tickets bought in last 12 months	Number of ticket orders in last 12 months	Average price of tickets bought in last 12 months	Total value of all tickets bought in last 12 months	Munich resident [yes$\,=\,$1]	Year of last ticket purchase [2006$\,=\,$1]
		(1)	(2)	(3)	(4)	(5)	(6)	(7)	(8)
1	Lead donor (control)	3770	.478	6.27	2.22	86.3	423	.416	.574
			(.008)	(.153)	(.046)	(.650)	(7.73)	(.008)	(.008)
2	Lead donor $+$ 1:.5 match	3745	.481	6.39	2.20	86.8	432	.416	.576
			(.008)	(.184)	(.049)	(.660)	(9.63)	(.008)	(.008)
			[.818]	[.606]	[.851]	[.603]	[.451]	[.989]	[.863]
3	Lead donor $+$ 1:1 match	3718	.477	6.46	2.28	85.8	435	.419	.576
			(.008)	(.148)	(.050)	(.667)	(9.78)	(.008)	(.008)
			[.923]	[.362]	[.329]	[.642]	[.314]	[.838]	[.890]
4	Lead donor $+$ 1:1 match for donations greater than €50	3746	.476	6.31	2.21	85.2	419	.426	.567
			(.008)	(.145)	(.046)	(.657)	(7.39)	(.008)	(.008)
			[.825]	[.832]	[.949]	[.238]	[.726]	[.399]	[.540]
5	Lead donor $+$ €20 match for any donation	3746	.486	6.09	2.20	86.5	416	.428	.556
			(.008)	(.132)	(.047)	(.657)	(8.05)	(.008)	(.008)
			[.525]	[.404]	[.765]	[.855]	[.578]	[.281]	[.108]

Mean, standard error in parentheses, *P* value on test of equality of means with control group in brackets. The tests of equality are based on an OLS regression allowing for robust standard errors. All monetary amounts are measured in Euros. The “last twelve months” refers to the year prior to the mail out from June 2005 to June 2006

Table 2 provides descriptive evidence on behavior on the intensive and extensive margins of charitable giving by treatment. For each statistic, we report its mean, its standard error in parentheses, and whether it is significantly different from that in the control treatment. Figure 1 provides a graphical representation of the outcomes across treatments, showing for each treatment *t* the average bundle chosen, $\mathbf {x}^{t}$, at the relevant price vector, $\mathbf {p}^{t}$. In our sample of 18,725 individual recipients, Columns 1–3 reveal that overall, 780 individuals donated a total of €75,350, corresponding to €116,489 raised for the project, with a mean donation given of €96.6.

**Table 2:** Outcomes by treatment-descriptive evidence

Treatment number	Treatment description	Comparison group	Total amount donated	Total amount raised	Number of donors	Response rate	Average donation received	Median donation received	Average donation given	Median donation given
			(1)	(2)	(3)	(4)	(5)	(6)	(7)	(8)
1	Lead donor (control)		17,416	17,416	132	.035	132	100	132	100
						(.003)	(14.3)		(14.3)	
2	Lead donor $+$ 1:.5 matching		15,705	23,558	156	.042	151	75	101	50
						(.003)	(18.9)		(12.6)	
		T1				[.134]	[.421]	[.131]	[.102]	[.000]
3	Lead donor $+$ 1:1 matching		14,310	28,620	155	.042	185	100	92.3	50
						(.003)	(20.7)		(10.4)	
		T1				[.133]	[.037]	[.999]	[.025]	[.000]
		T2				[.994]	[.231]	[.217]	[.609]	[1.000]
4	Lead donor $+$ 1:1 matching for donations greater than €50		15,671	31,107	160	.043	194	120	97.9	60
						(.003)	(19.3)		(9.59)	
		T1				[.084]	[.010]	[.102]	[.049]	[.000]
		T2				[.820]	[.109]	[.001]	[.863]	[.149]
		T3				[.826]	[.730]	[.260]	[.681]	[.260]
5	Lead donor $+$ €20 match for any donation		12,248	15,788	177	.047	89.2	70	69.2	50
						(.003)	(5.51)		(5.51)	
		T1				[.008]	[.006]	[.065]	[.000]	[.002]
		T2				[.240]	[.002]	[.751]	[.023]	[1.000]
		T3				[.244]	[.000]	[.008]	[.049]	[1.000]
		T4				[.343]	[.000]	[.000]	[.010]	[.084]

Mean, standard error in parentheses. *P* values on tests of equalities on means with comparison group in brackets. The test of equality of means is based on an OLS regression allowing for robust standard errors. The test of equality of medians is based on a quantile regression. The total amount raised corresponds to the sum of donations of all individual recipient observations. The response rate is the proportion of recipients that donate some positive amount, as reported in the donation amount column. The actual donation then received by the opera house in each treatment is reported in the donation received column. All monetary amounts are measured in Euros

On the extensive margin of giving, Column 4 shows that response rates vary from 3.5 to 4.7% across treatments, which are almost double those in comparable large-scale natural field experiments on charitable giving (Eckel and Grossman 2008; Karlan and List 2007). Indeed, a rule of thumb used by charitable organizations is to expect response rates to mail solicitations of between .5 and 2.5% (De Oliveira et al. 2011).

On the relative price of giving we note that despite there being large variations in the budget sets in treatments T1–T3, there are no statistically significant differences in response rates across these treatments. On the intensive margin, Column 5 shows that in the control treatment T1, the average donation given is €132. As the relative price of donations received falls in treatments T2 and T3, the average donation received increases to €151 in T2 with a 50% match rate, and to €185 in T3 with a 100% match rate. As shown in Fig. 1 and Column 7 of Table 2, as the match rate increases, the average donation *given*, $d_{g}$, falls from €132 in the control treatment T1 to €101 in T2 with a 50% match rate, and to €92.3 in T3 with a 100% match rate.

Treatment T4 induces recipients to face a non-convex budget set. For donations below €50, the budget line is coincident with that of the control treatment T1, for donations at or above €50, it coincides with that of the 100% matching treatment T3. Figure 1 shows that average outcome in terms of donations given and received in T4 replicate almost exactly those in the 100% matching treatment T3—the average donation received in T4 is €194, as opposed to €185 in T3, and the average donation given is €97.9, as opposed to €92.3 in T3. To see why this is so, note that in the control treatment, the average donation received is €132. This suggests the portion of the budget line in T4 that lies to the left of €100 on the *x*-axis of donations received is irrelevant for many recipients. In essence, treatments T3 and T4 present the average recipient with an almost identical choice. Hence, response rates and donations should not differ markedly between the two.

Treatment T5—that causes a parallel shift out of the budget set conditional on any positive donation—should induce the largest change in the number of donors relative to the control group, because *any* individual with preferences, such that $\left. MRS_{c,d_{r}}\right| _{d_{r}=0}<0$ will find it optimal to donate some amount in T5, whereas this is not the case in other treatments. The response rate is, indeed, significantly higher in T5 relative to the other treatments. However, it is still only 4.7%, highlighting that even among this targeted population, 95% of individuals do not care for the project. Comparing the income treatment T5 to the control treatment, consumer theory suggests that these additional donors should be willing to contribute relatively small amounts to the project which is strongly supported in the data.

## Testing revealed preference theory

### Aggregate violations

As the budget sets in treatments T1 to T5 intersect or overlap, as shown in Fig. 1, pairwise comparisons of the average behavior of individuals in any two treatments lead to tests of whether behavior is consistent with revealed preference theory. These tests are of three types: (i) the proportion of recipients that should donate some positive amount; (ii) the proportion of recipients that lie above or below some critical threshold, which is typically where the two budget lines intersect; and (iii) the distribution of donations given and received.

An example of the first type of test is given by comparing treatments T1 and T3. As shown in Fig. 1, the budget set expands moving from T1 to T3. Assuming that individual preferences are well behaved, the proportion of individuals that find it optimal to provide some positive donation under T3 should be at least as great as the proportion that respond under T1.

An example of the second type of test is given by comparing treatments T2 and T5 in which the budget sets cross at donations given equal to €40. For all donations given greater than €40, the budget set expands under T2 relative to T5. Hence, revealed preference arguments imply the proportion of donations given that are at least €40 should be weakly higher in T2 than T5.

An example of the third type of test is given by comparing treatments T3 and T4. As shown in Fig. 1, the budget sets are coincident for donations given that are more than €50. Hence, the distribution of donations given conditional on them being more than €50, should be identical in both treatments. This follows from the fact that any donors that contribute strictly more than €50 under T3 should, by revealed preference, also contribute the same under T4.

Table 3 presents the results for each pairwise treatment comparison. Columns (1)–(3) give the hypotheses to be tested of the type: ”the behavior is consistent with revealed preferences.” One test is boxed as it requires the additional assumption of strict convexity in addition to satisfying GARP. For each test, we report the *p* value on the null hypothesis consistent with revealed preference theory. Thirteen of the fourteen tests do not reject the hypothesis that consumers, on average, having an underlying utility function that displays standard properties.

**Table 3:** Pairwise tests of revealed preference

Treatments being compared		Type of comparison	Response rate [one-sided *t* test]	Proportions above/below some critical value [one-sided *t* test]		Distribution of donations given [Mann–Whitney test]
			(1)	(2)		(3)
T1: lead donor (control)	T2: lead donor + 1:.5 match	Budget set expands	Weakly higher in T2			
			[.933]			
T1: lead donor (control)	T3: lead donor + 1:1 match	Budget set expands	Weakly higher in T3			
			[.934]			
T1: lead donor (control)	T4: lead donor + 1:1 match for donations greater than €50	Budget set expands and partly coincides	Weakly higher in T4			
			[.958]			
T2: lead donor + 1:.5 match	T4: lead donor + 1:1 match for donations greater than €50	Budget sets cross		Proportion of donations < 50 weakly higher in T2	Proportion of donations > 50 weakly higher in T4	
				[1.000]		
T2: lead donor + 1:.5 match	T5: lead donor + €20 match for any donation	Budget sets cross	Weakly higher in T5	Proportion of donations < 40 weakly higher in T5	Proportion of donations > 40 weakly higher in T2	
			[.880]	[.986]		
T3: lead donor + 1:1 match	T4: lead donor + 1:1 match for donations greater than €50	Budget set expands and partly coincides	Weakly higher in T3			Identical for donations > 50 (if no focal point effects)
			[.413]			[.000]
T3: lead donor + 1:1 match	T5: lead donor + €20 match for any donation	Budget sets cross	Weakly higher in T5	Proportion of donations < 20 weakly higher in T3	Proportion of donations > 20 weakly higher in T5	
			[.878]	[.988]		
T4: lead donor + 1:1 match for donations greater than €50	T5: lead donor + €20 match for any donation	Budget sets cross	Weakly higher in T5	Proportion of donations < 50 weakly higher in T5	Proportion of donations > 50 weakly higher in T4	
			[.828]	[1.000]		

Hypotheses being tested in columns (1)–(3). They describe behavior that is, on average, consistent with revealed preferences. *P* value on relevant test in brackets below. The test in the column (3) requires the assumption of convexity on consumer preferences. The tests of proportions are based on all mail out recipients

The exception is the test between T3 and T4 in the last column that is based on the assumption of convexity. To examine this violation in more detail, we note that if preferences are convex, then by revealed preference, individuals who would have donated less than €50 in T3 are expected to donate no more than €50 in T4. Hence, relative to T3, there ought to be relatively more donations given *below or at* $d_{g}=$ €50 in T4. In the data there is, however, a bunching of donations in T4 relative to T3 slightly above $d_{g}=$ €50, and a fall in the proportion of donations given below €50, that is, we find that donors prefer to give incrementally above €50 when faced with the non-convex budget set (perhaps to avoid the appearance of being “cheap”).

### Individual violations

In our between-subject design, we do not observe the same consumer making multiple choices under alternative budget sets. To detect *individual* violations of GARP, we propose a novel approach based on the estimate for each individual *i*, whose actual choice we only observe in treatment *t*, for what she would have donated in the relevant counterfactual treatment $$t^{\prime }\ne t$$ based on the predictions from a hurdle model. This takes explicit account of the fact that the initial decision to donate ($$D_{i}=0$$ or 1) may be separated from the decision of how much to donate: the choice of $$d_{r}$$ conditional on $$ D_{i}=1$$. A simple two-tiered model for charitable giving has, as a first stage, a probit model of giving. At the second stage, we assume that donations received from individual *i* are log normally distributed conditional on $$ d_{ri}>0$$. The maximum-likelihood estimator of the second-stage parameters is then simply the OLS estimator from the following regression:

2 $$\begin{aligned} \log (d_{ri})=\beta T_{i}+\gamma X_{i}+z_{i} \quad \text { for }\;d_{ri}>0, \end{aligned}$$

where $T_{i}$ is a dummy for any treatment $T_{i}$ that the individual was assigned to (T2–T5). We estimate the coefficients relative to a control treatment for each treatment separately.^3^ We also control for the following individual characteristics $X_{i}$, to reduce the sampling errors of the treatment effect estimates: whether recipient *i* is female, the number of ticket orders placed in the 12 months prior to mail out, the average price of these tickets, whether *i* resides in Munich, and a dummy for whether the year of the last ticket purchase was 2006. We calculate robust standard errors. More details of the procedure are provided in the Technical Appendix.

In a second step, for each individual and treatment that this individual was not in, we predict her donation amount based on her individual characteristics, fictive treatment assignment, and the coefficient estimates from the first stage. We use this comparison between one actual treatment *t* and one predicted counterfactual treatment $t^{\prime }$ as the basis of tests for individual violations of revealed preference theory.^4^ There are 10 such pairwise comparisons, as shown in Table 4. These are analogous to a subset of the tests performed in Table 3, namely those for which the budget sets intersect. Column 1 shows the number of violations of revealed preference theory for each pairwise comparison of treatments. We also show the proportion of violations defined as the number of violations divided by the number of positive actual donations that fulfill the first part of the condition.^5^ Both measures have been previously used in the literature as measures of goodness of fit in tests of revealed preference (Gross 1995).

**Table 4:** Individual violations of revealed preference

Matching treatments being compared		Type of comparison	GARP violation	Number (percentage) of violations	Donation given among violators [95% confidence interva]	Number (percentage) of violations, predicted high donors	Alternative hypothesis: number (percentage) of violations
				(1)	(2)	(3)	(4)
T1: lead donor (control)	T4: lead donor $+$ 1:1 match for donations greater than €50	Budget set expands and partly coincides	Give more than 50 in T1 [$N=70$] and predicted to give less than 50 in T4	1	49.5		1
				1.4%			1.4%
			Give less than 50 in T4 [$N=11$] and predicted to give more than 50 in T1	3	52.3		8
				27.3%	[44.8, 59.7]		72.7%
T2: lead donor $+$ 1:.5 match	T4: lead donor $+$ 1:1 match for donations greater than €50	Budget sets cross	Give more than 50 in T2 [$N=62$] and predicted to give less than 50 in T4	2	48.2		2
				3.2%	[38.4, 58.0]		3.2%
			Give more than 50 in T4 [$N=128$] and predicted to give less than 50 in T2	14	44.8		35
				10.9%	[42.0, 47.6]		27.3%
T2: lead donor $+$ 1:.5 match	T5: lead donor $+$ €20 match for any donation	Budget sets cross	Give less than 40 in T2 [$N=48$] and predicted to give more than 40 in T5	46	68.0	23	37
				95.8%	[63.0, 73.0]	47.92%	77.1%
			Give more than 40 in T5 [$N=103$] and predicted to give less than 40 in T2	0	–	0	7
				0.0%		0.0%	6.8%
T3: lead donor $+$ 1:1 match	T5: lead donor $+$ €20 match for any donation	Budget sets cross	Give less than 20 in T3 [$N=15$] and predicted to give more than 20 in T5	15	62.3	3.00	15
				100.0%	[53.9, 70.7]	20.00%	100.0%
			Give more than 20 in T5 [$N=132$] and predicted to give less than 20 in T3	0	–	0.00	0
				0.0%		0.0%	0.0%
T4: lead donor $+$ 1:1 match for donations greater than €50	T5: lead donor $+$ €20 match for any donation	Budget sets cross	Give less than 50 in T4 [$N=11$] and predicted to give more than 50 in T5	10	64.0	3.00	3
				90.9%	[57.8, 70.1]	27.3%	27.3%
			Give more than 50 in T5 [$N=55$] and predicted to give less than 50 in T4	0	–	0.00	0
				0.0%		0.0%	0.0%

The number of violations is based on recipients that responded with some positive donation in their assigned treatment. The percentage of violations is the number of violations divided by the number of individuals that fulfills the first part of the condition (*N* given in square brackets). In Columns 1 and 4, the proportion of violations is the number of violations divided by the total number of positive donations given in the treatment from which actual (and not predicted) donations are used. Column 2 shows the predicted donation in each pairwise comparison among those individuals that violate the predictions of revealed preference theory. The pairs in Column 3 are restricted to those that are predicted to give higher than average amounts (absent any match). In Column 4, we form predicted donations by regressing the log of donations received on observable characteristics of the recipient but not the treatment dummy

Across pairwise comparisons, the proportion of violations varies. To provide a sense of the magnitude of such violations, Column 2 shows the average donation given *among* violators of GARP and a 95% confidence interval. The first row shows that individuals that violate GARP and donate less than €50 in T4, on average, actually donate €49.5. Hence, there are a small number of violations of this prediction of revealed preference theory, and the magnitude of the violations is small. In contrast, the fifth row shows that individuals that violate GARP and donate more than €40 in T5, on average, actually donate €68. Hence, for this test, there are both a relatively large number of violations and those violations are quantitatively large.

For comparisons involving the income treatment T5, Column 3 restricts the sample to high valuation recipients who, based on their predicted donation from (2), would likely donate more than €20 even absent any match, to avoid confounding the comparisons with a change in the identity of the marginal donor. For these donors, the treatment corresponds to a * de facto* increase in income rather than a conditional increase in income as they would have donated some positive amount in any case. When focusing on high valuation donors, the number of violations falls considerably. This highlights that some of the earlier violations are likely driven by changes in the composition of donors across treatments. In particular, there are likely to be low valuation donors that give positive amounts in the income treatment T5 but that would not have donated in any other counterfactual treatment.

To summarize, the behavior of 88 individuals is predicted to violate revealed preferences (out of 466),^6^ while at least 80% of recipients’ behavior is consistent with GARP. Whether this is a large or small number depends on the power of our tests, which, in turn, requires a specific alternative hypothesis to be specified (Varian 1982; Bronars 1995). On the one hand, in contrast to non-experimental methods, our field experiment allows us to engineer large changes in relative prices holding everything else equal. This improves the power of our test. On the other hand, the bundle at which the budget sets intersect in any two treatments in our design is distant from the bundle chosen on average in the treatments, thus lowering the power of our test. The extent to which these factors offset one another varies across each of the pairwise comparisons in Table 4.

To provide a sense of which of the pairwise comparisons are most informative, we consider the following alternative hypothesis. We generate predicted choices for each donor by first estimating a specification analogous to (2) but excluding the treatment dummy. Column 4 of Table 4 then shows the number and percentage of violations of GARP that would have occurred under this alternative hypothesis. For eight out of the ten pairwise comparisons, the number of actual violations is equal or smaller than the number of violations based on this alternative, in some cases by orders of magnitudes, suggesting that these pairwise comparisons are powerful tests of GARP. More details of this test are provided in the Technical Appendix.

## Conclusions

We have presented evidence from the first large-scale natural field experiment designed to shed light on whether consumer behavior is consistent with the predictions of revealed preference theory. We do so in the context of a field experiment on charitable giving which allows us to vary budget sets experimentally in a straightforward and very natural manner. We find that consumer behavior, on both the extensive and intensive margins of charitable giving, can be rationalized within a standard model of consumer choice in which individuals have preferences over their own consumption and their contribution towards the charitable project. The behavior of at least 80% of recipients is in line with them adhering to GARP. In short, in a real-world static environment where participants make simple decisions they are familiar with, the predictions of microeconomic theory work well in explaining the observed choices of individuals.

## Electronic supplementary material

Below is the link to the electronic supplementary material.

Supplementary material 1 (pdf 330 KB)

## Footnotes

1 Our results differ from some of the laboratory evidence on consumer choice, such as Battalio et al. (1973) and Sippel (1997) who find behavior not to be in line with GARP. This may be because, in our study, consumers are faced with a real-life setting and make simple decisions which they are familiar with, and we exploit a large sample of individuals.

2 Our analysis here focuses on the broad question of whether individual behavior is consistent with neoclassical microeconomic theory. In companion papers, we exploit the natural field experiment to shed light on specific issues relating to the economics of charitable giving (Huck and Rasul 2011; Huck et al. 2015).

3 The omitted treatment is T1 for T2–T5 and a treatment T0 without a lead donor for T1.

4 We do not compare predicted choices with each other.

5 Notice that an alternative would be to take the entire sample as a denominator (for example, people who always give zero are always consistent). Our more conservative approach adjusts for cases of low power.

6 Note that some conditions overlap.
