# Supplementary material for: Testing consumer theory: evidence from a natural field experiment
Source: J Econ Sci Assoc. 2017 Nov 28;3(2):89–108. doi: 10.1007/s40881-017-0040-3 (PMC6956945; doi:10.1007/s40881-017-0040-3)
Supplement: Supplementary file 1 — Supplementary material 1 (pdf 330 KB) [file 40881_2017_40_MOESM1_ESM.pdf]

Technical Appendix to: TESTING CONSUMER THEORY: EVIDENCE FROM A NATURAL FIELD EXPERIMENT

MAJA ADENA (WZB) STEFFEN HUCK (WZB & UCL)
IMRAN RASUL (UCL)

SEPTEMBER 2017

Abstract

This appendix contains some additional details of the analysis presented in section 4.2 in the main paper and an additional robustness test. It also includes the mail out letter and its translation.

1 The Prediction Model

It is important to be clear that by using a between-subject design and therefore having to predict recipient behavior to treatments they were not assigned to, the test of GARP partly relies on the accuracy of the prediction model. Table T1 presents the estimates from model (2) that form base for our predictions. Note that the regressions are run pairwise, relative to the control treatment. Out of six regressions only one of the treatment coefficients (on T4) is not significant but it is, at the same time, very small compared to the others.

The potential inaccuracy of the prediction model raises a number of issues. First, it would always be preferable to control for a rich a set of covariates as possible. In this setting, we are restricted to those observables available from the opera house's database that relate to proxies of individual affinity to the opera house and income. Future field experiments should engage in more primary data collection to help address this issue.

Second, the prediction has some associated standard error. Hence although the point estimate of the prediction may suggest a lack of violation of GARP, or *vice versa*, this might not be a particularly informative statement. To see the precision of the prediction, Column 2 also gives the 95% confidence interval for predicted donations among violators of GARP. For example, for the third test based on a comparison of actual behavior in T2 and predicted behavior in T4, the point estimate on the prediction of violators is €48.2 and the 95% confidence interval is from €38.4 to €58. As the confidence interval runs above €50, it might be that all violators actually adhere to GARP. Of course the prediction error also implies that some individuals assigned to adhere to GARP according to their point estimate, might not actually do so at conventional significance levels. In this regard, it is probably helpful to focus on the behavior of a relatively homogeneous group: a specifically targeted group of opera goers, and among this group, those that actually choose to make some positive donation.

Third, the prediction model might be misspecified. As a robustness check, we note that allowing for heterogenous treatment effects in each treatment, so that the predicted donation is based on a regression in which each characteristic is also interacted with the relevant treatment dummy, yields similar conclusions. Clearly, more sophisticated approaches could be applied to make predictions that are more robust to functional form misspecifications such as the non-parametric methods developed in Blundell *et al* [2003, 2007].

2 Power

The second set of issues to be discussed with this methodology is in regard to the power of the test. Whether 80% of non-violations of GARP is considered a large or small number depends on the power of our tests, which in turn requires a specific alternative hypothesis to be specified [Varian 1982, Bronars 1995, Andreoni and Harbaugh 2008]. On the one hand, in contrast to non-experimental methods, our field experiment allows us to engineer large changes in relative

prices holding everything else equal. This improves the power of our test. In addition we note that the observables controlled for do have some predictive power in explaining the variation in donations. More precisely we find that in general, recipients that have placed more ticket orders in the 12 months prior to the mail out, and have paid a higher average price per ticket over the same period, donate significantly more regardless of whichever treatment they are assigned to. Hence the observables we condition explain some of the variation in donations, increasing the power of our test to detect violations of revealed preference theory, all else equal.

On the other hand, the bundle at which the budget sets intersect in any two treatments in our design is distant from the bundle chosen on average in the treatments, thus lowering the power of our test. The extent to which these factors offset one another varies across each of the pairwise comparisons in Table 4, but this is a shortcoming of our design that should be borne in mind for the results. This does not however detract from the methodological contribution of our analysis that field experiments can be crafted to test revealed preference theory.[1]

To provide a sense of which of the pairwise comparisons therefore are most informative, we consider the following alternative hypothesis. We generate predicted choices of each donor by first estimating a specification analogous to (2) but not controlling for any treatment dummy, including the omitted control treatment T1. The results are provided in Table T2. Hence under this alternative we assume donations are driven purely by the observables listed in Table 1 rather than treatment assignment. Column 4 of Table 4 then shows the number and percentage of violations of GARP that would have occurred under this particular alternative hypothesis.

For eight out of the ten pairwise comparisons—except row 5 and 9—the number of violations based on this alternative are always at least as large as the actual number of violations. Note that if the number (percentage) of violations based on the alternative is small, there is not much room for improvement—like in row 10. In some cases, the number of actual violations is orders of magnitude smaller than would be expected from this alternative hypothesis, suggesting these pairwise comparisons are powerful tests of GARP. For example, in the comparison between observed donations in T4 and predicted donations in T2, the actual number of violations is 14 while 35 violations are predicted under the alternative hypothesis. Similarly, comparing observed donations in T5 and predicted donations in T2, the actual number of violations is 0 while 7 violations are predicted under the alternative hypothesis.

In contrast, a few of the other comparisons distinctly lack power against this specific alternative, which is as expected given the shortcomings arising from the precise location of intersection between budget sets described above. For example, the comparison of actual behavior in T5 to predicted behavior in T3 yields zero violations of GARP under both our test and this alternative hypothesis, so this particular comparison is not informative of whether individual behavior can be rationalized by GARP in this setting. These findings highlight that although the methodological approach of using a field experiment to test for GARP has many advantages over laboratory

[1] A series of indices of power of GARP tests are presented in Andreoni and Harbaugh [2008].

or non-experimental approaches, the mere fact that large price changes can be induced is not sufficient to guarantee that tests of GARP have high power against an alternative hypothesis.

Although both methodological issues—the accuracy of the prediction model and power of the GARP test—apply to all empirical approaches, solutions to both might more readily available to field experimenters. In future work using this approach, experimenters need to engage in data collection and design interventions that improve the accuracy and robustness of the prediction model, and allow for more powerful tests of GARP by engineering budget line intersections at bundles closer to the expected behavior of more individuals.

3 Focal Point Effects?

The results in Table 4 highlight that the pairwise comparisons that yield the highest percentage of violations all involve the non-convex treatment T4. As discussed earlier, this might be because individuals that would have given less than €50 in T1 choose to donate slightly more than revealed preference theory predicts in T4 and so do locate just above the interior corner solution of €50 in T4. Moreover, the wording in T4 in the mail out letter might lead to d_g = €50 becoming focal for recipients. If so, then relative to T3 there ought to be bunching in the distribution of donations given *from above* at d_g = €50 in T4. No such bunching above d_g = €50 is predicted in the standard model of consumer choice—this segment of the budget line is available under both T3 and T4.

To explore we use quantile regression methods to characterize the effect of being assigned to treatment T4 relative to T3 on different percentiles of the conditional distribution of donations given, d_g. This allows us to estimate changes in the shape and spread of the conditional distribution of donations given, not just the change in the mean as estimated in (2). We therefore estimate the following quantile regression specification at each quantile $\theta \in [0, 1]$,

$$Quant_{\theta}(\log(d_{gi})|.) = \beta_{\theta} T4_i + \gamma_{\theta} X_i \text{ for } d_{gi} > 0. \quad (1)$$

The parameter of interest, β_{θ}, measures the difference at the θth conditional quantile of log donations received between the treatment group T4 relative to the omitted group T3. Figure 2B graphs estimates of β_{θ} from (1) and the associated 95% confidence interval at each quantile. We also show the quantile that corresponds to donations given of €50 (€60) across T3 and T4.

Figure T2 shows the distribution of donations given becomes less dispersed in T4 and in particular, this is because there is a bunching of donations given *just above* €50 in T4 relative to T3. The estimates show that there are recipients that would otherwise have given less than €50 in T3 are those that shift their donations towards €50 and slightly above. There is no evidence that recipients who would have otherwise given above €50 significantly reduced their donations towards €50 in T4. Indeed, the distribution of donation given is little changed above €70 in line

with there being no or very weak focal point effects introduced by the non-convex treatment.[2]

References

[1] ANDREONI.J AND W.T.HARBAUGH (2008) Power Indices for Revealed Preference Tests, mimeo, UCSD.

[2] BLUNDELL.R, M.BROWNING, AND I.CRAWFORD (2003) "Nonparametric Engel Curves and Revealed Preference", *Econometrica* 71: 205-40.

[3] BLUNDELL.R, M.BROWNING, AND I.CRAWFORD (2007) "Improving Revealed Preference Bounds on Demand Responses", *International Economic Review* 48: 1227-44.

[4] BRONARS.S.G (1995) "The Power of Nonparametric Tests of Preference Maximisation", *Econometrica* 55: 693-98.

[5] IYENGAR.S AND M.LEPPER (2000) "When Choice is Demotivating: Can One Have Too Much of a Good Thing?", *Journal of Personality and Social Psychology* 79: 995-1006.

[6] VARIAN.H.R (1982) "The Nonparametric Approach to Demand Analysis", *Econometrica* 50: 945-74.

[2]The analysis highlights that by removing a portion of the budget set for donations given less than €50 in T4 relative to T3, most small donors optimally move to the interior corner solution rather than the exterior solution, while large donors are unaffected—the response rates are almost identical in treatments T3 and T4. This is in contrast to some findings in the psychology literature, where consumers are sometimes observed forgoing a decision altogether in the presence of an expanded choice set [Iyengar and Lepper 2000].

Appendix: The Mail Out Letter (Translated)

Bayerische Staatsoper
Staatsintendant
Max-Joseph-Platz 2, D-80539 München
www.staatsoper.de

[ADDRESS OF RECIPIENT]

Dear [RECIPIENT],

The Bavarian State Opera House has been investing in the musical education of children and youths for several years now as the operatic the art form is in increasing danger of disappearing from the cultural memory of future generations.

Enthusiasm for music and opera is awakened in many different ways in our children and youth programme, “Erlebnis Oper” *[Experience Opera]*. In the forthcoming season 2006/7 we will enlarge the scope of this programme through a new project “Stück für Stück” that specifically invites children from schools in socially disadvantaged areas to a playful introduction into the world of opera. Since we have extremely limited own funds for this project, the school children will only be able to experience the value of opera with the help of private donations.

[This paragraph describes each matching scheme and is experimentally varied as described in the main text of the paper].

As a thank you we will give away a pair of opera tickets for Engelbert Humperdinck’s “Konigskinder” on Wednesday, 12 July 2006 in the music director’s box as well as fifty CDs signed by Maestro Zubin Mehta among all donors.

You can find all further information in the enclosed material. In case of any questions please give our Development team a ring on *[phone number]*. I would be very pleased if we could enable the project “Stück für Stück” through this appeal and, thus, make sure that the operatic experience is preserved for younger generations.

With many thanks for your support and best wishes,

Sir Peter Jonas, Staatsintendant

Appendix: The Mail Out Letter (Translated)

“Stück für Stück”

The project “Stück für Stück” has been developed specifically for school children from socially disadvantaged areas. Musical education serves many different functions in particular for children and youths with difficult backgrounds -- it strengthens social competence and own personality, improves children’s willingness to perform, and reduces social inequality. Since music education plays a lesser and lesser role in home and school education, the Bavarian State Opera has taken it on to contribute to it ourselves. The world of opera as a place of fascination is made attainable and accessible for young people.

In drama and music workshops, “Stück für Stück” will give insights into the world of opera for groups of around 30 children. They will be intensively and creatively prepared for a subsequent visit of an opera performance. These workshops encourage sensual perception – through ear and eye but also through scenic and physical play and intellectual comprehension – all of these are important elements for the workshops. How does Orpheus in “Orphee and Eurydice” manage to persuade the gods to let him save his wife from the realm of dead? Why does he fail? Why poses the opera “Cosi fan tutte” that girls can never be faithful? It is questions like these that are investigated on the workshops.

The workshops are also made special through the large number and variety of people who are involved in them: musicians, singers, directors, and people from many other departments, ranging from costumes and makeup to marketing. The participants in each workshop work through an opera’s storyline, and are introduced to the production and will meet singers in their costumes as well as musicians. This makes the workshops authentic. After the workshops the participants are invited to see the actual opera production.

Through your donation the project “Stück für Stück” will be made financially viable so that we can charge only a small symbolic fee to the participants. This makes it possible to offer our children and youth programme also to children from socially disadvantaged backgrounds that can, thus, learn about the fascination of opera.

Note: In German, Stück für Stück is a wordplay --- “Stück” meaning “play” as in drama and “Stück für Stück” being an expression for doing something bit by bit.

Table T1: Estimates of the Model (2)

Dependent variable: Donation given(a)

Baseline	T0	T1	T1	T1	T1	T1
Treatment	T1	T2	T3	T4	T5	T5 high donors
Treatment dummy	0.366***	-0.308**	-0.323***	-0.054	-0.505***	-0.324***
	(0.122)	(0.134)	(0.120)	(0.107)	(0.117)	(0.104)
Female dummy	0.066	-0.203	0.111	-0.046	-0.032	0.094
	(0.124)	(0.131)	(0.120)	(0.107)	(0.113)	(0.103)
Number of ticket orders in last 12 months	0.028**	0.037***	0.028**	0.014	0.028**	0.030**
	(0.013)	(0.014)	(0.011)	(0.011)	(0.013)	(0.013)
Average value of tickets	0.007***	0.009***	0.008***	0.007***	0.008***	0.007***
	(0.001)	(0.002)	(0.001)	(0.001)	(0.002)	(0.001)
Munich postcode (yes=1)	0.102	0.192	0.161	0.143	0.206*	0.200*
	(0.136)	(0.132)	(0.122)	(0.106)	(0.117)	(0.107)
Dummy=1 if year of last ticket purchase=2006, =0 if earlier	0.117	0.219	0.027	0.128	0.231*	0.044
	(0.146)	(0.153)	(0.146)	(0.129)	(0.130)	(0.116)
Constant	2.987***	3.143***	3.288***	3.518***	3.219***	3.593***
	(0.220)	(0.253)	(0.209)	(0.222)	(0.228)	(0.201)
Observations	274	288	287	292	309	251
Adjusted R^2	0.103	0.114	0.158	0.077	0.149	0.131

Notes: Robust standard errors in parentheses; * $p < 0.10$, ** $p < 0.05$, *** $p < 0.01$, (a) the dependent variable is transformed as follows: f(X)=ln(X-k), with k chosen such that the distribution is symmetric around the mean.

Table T2: Estimates of the Model (2) without treatment dummies

Dependent variable: Donation given(a)

Baseline	T0	T1	T1	T1	T1
Treatment	T1	T2	T3	T4	T5
Female dummy	0.115	-0.182	0.143	-0.047	-0.004
	(0.126)	(0.131)	(0.120)	(0.107)	(0.116)
Number of ticket orders in last 12 months	0.032**	0.039***	0.031***	0.015	0.032**
	(0.013)	(0.014)	(0.011)	(0.011)	(0.012)
Average value of tickets	0.007***	0.009***	0.009***	0.007***	0.009***
	(0.001)	(0.002)	(0.001)	(0.001)	(0.002)
Munich postcode (yes=1)	0.094	0.197	0.149	0.137	0.159
	(0.139)	(0.133)	(0.123)	(0.105)	(0.120)
Dummy=1 if year of last ticket purchase=2006, =0 if earlier	0.134	0.249	0.020	0.129	0.275**
	(0.147)	(0.151)	(0.145)	(0.129)	(0.132)
Constant	3.102***	2.921***	3.094***	3.487***	2.858***
	(0.222)	(0.218)	(0.199)	(0.208)	(0.209)
Observations	274	288	287	292	309
Adjusted R2	0.075	0.099	0.139	0.080	0.096

Notes: Robust standard errors in parentheses; * $p < 0.10$, ** $p < 0.05$, *** $p < 0.01$, (a) the dependent variable is transformed as follows: f(X)=ln(X-k), with k chosen such that the distribution is symmetric around the mean.

Figure T1: Non Convex Budget Set T4 Relative to 100% Match Rate Treatment T3

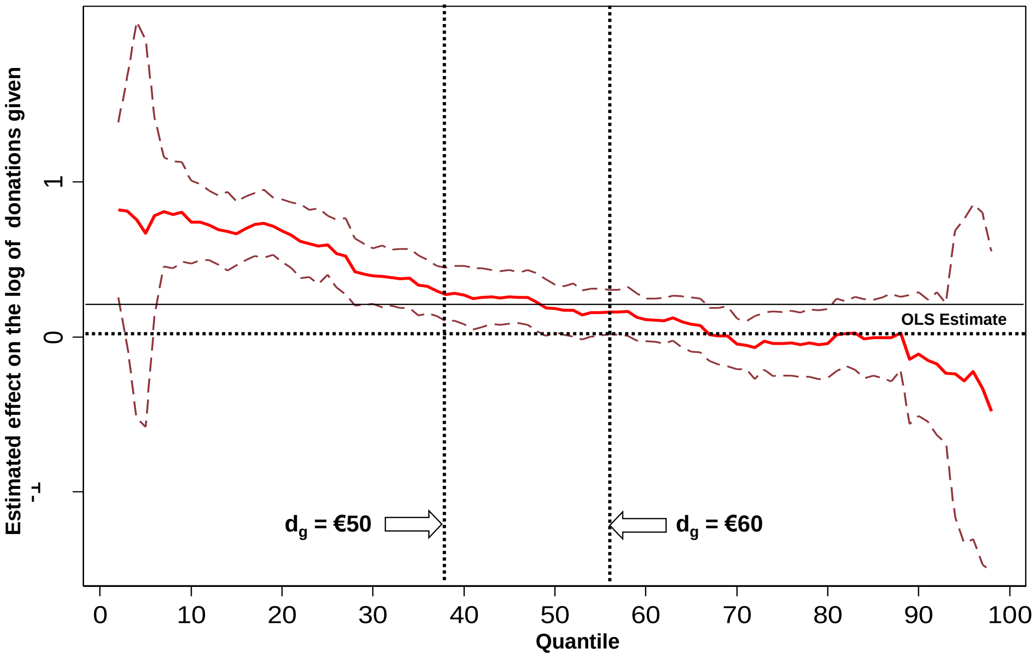

Notes: The figure shows the estimated effect of being assigned to the non-convex treatment T4 relative to being assigned to the 100% matching treatment T3 on the log of donations given, at each quantile of the conditional distribution of the log of donations given, and the associated 95% confidence interval. The figure also shows the coefficient on the treatment dummy variable from an OLS regression. The individual characteristics controlled for are whether the recipient is female, the number of ticket orders placed in the 12 months prior to mail out, the average price of these tickets, whether the recipient is a Munich resident, and a dummy variable for whether the year of the last ticket purchase was 2006 or not.
